# Analysis of cancer metabolism with high-throughput technologies

**DOI:** 10.1186/1471-2105-12-S10-S8

**Published:** 2011-10-18

**Authors:** Aleksandra A Markovets, Damir Herman

**Affiliations:** 1 Department of Information Science, UALR/UAMS Joint Graduate Bioinformatics Program, University of Arkansas, Little Rock, AR, 72204, USA; 2 Department of Internal Medicine, Division of Hematology and Oncology, Winthrop P. Rockefeller Cancer Institute, University of Arkansas for Medical Sciences, Little Rock, AR, 72205, USA

## Abstract

**Background:**

Recent advances in genomics and proteomics have allowed us to study the nuances of the Warburg effect – a long-standing puzzle in cancer energy metabolism – at an unprecedented level of detail. While modern next-generation sequencing technologies are extremely powerful, the lack of appropriate data analysis tools makes this study difficult. To meet this challenge, we developed a novel application for comparative analysis of gene expression and visualization of RNA-Seq data.

**Results:**

We analyzed two biological samples (normal human brain tissue and human cancer cell lines) with high-energy, metabolic requirements. We calculated digital topology and the copy number of every expressed transcript. We observed subtle but remarkable qualitative and quantitative differences between the citric acid (TCA) cycle and glycolysis pathways. We found that in the first three steps of the TCA cycle, digital expression of aconitase 2 (*ACO2*) in the brain exceeded both citrate synthase (*CS*) and isocitrate dehydrogenase 2 (*IDH2*), while in cancer cells this trend was quite the opposite. In the glycolysis pathway, all genes showed higher expression levels in cancer cell lines; and most notably, digital gene expression of glyceraldehyde-3-phosphate dehydrogenase (*GAPDH*) and enolase (*ENO*) were considerably increased when compared to the brain sample.

**Conclusions:**

The variations we observed should affect the rates and quantities of ATP production. We expect that the developed tool will provide insights into the subtleties related to the causality between the Warburg effect and neoplastic transformation. Even though we focused on well-known and extensively studied metabolic pathways, the data analysis and visualization pipeline that we developed is particularly valuable as it is global and pathway-independent.

## Background

Profound differences between the metabolic pathways of normal and cancer cells have been known since 1926 when Otto Warburg attributed tumors to dysfunctional mitochondria [1]. Normal eukaryotic cells generate energy in the form of adenosine triphosphate (ATP) through a combination of glycolysis and the TCA pathway. The TCA pathway consists of enzymes encoded by 8 genes and the full cycle generates 36 molecules of ATP. On the other hand, glycolysis is a linear pathway that comprises enzymes encoded by 10 genes and produces only 2 molecules of ATP [2,3]. Under normal conditions glycolysis preferentially occurs in hypoxic circumstances yielding lactic acid [4]. In many types of cancer, glycolysis is the preferable method for meeting energy demands in cells undergoing uncontrolled growth even in the presence of oxygen [5]. This drastic metabolic shift is essentially the well-known Warburg effect [1]. While enzymes and substrates of the TCA cycle and glycolysis have been extensively studied, the causality between the Warburg effect and cancer remains unknown [6].

Improvements in ‘-omics’-based technologies, in particular genomics and proteomics, have allowed us to examine at a new level the molecular details of the Warburg effect. For example, it has been found that tumor glycolysis enhances activation of oncogene and loss of tumor suppressor gene activity by stabilization of the hypoxia-inducible factor (HIF), a transcription factor that regulates 9 out of 10 enzymes involved in glycolysis [5]. However, molecular differences between the TCA cycle and glycolysis and the impact of the expression of genes on rates and quantities of ATP production is currently unknown.

Metabolic pathways are regulated at several levels, including mRNA transcription, translation, and protein interaction [2]. We used next- (or second-) generation sequencing technology with RNA-Seq to study the TCA cycle and glycolysis at the transcriptional level of genes encoding their enzymes. This genomics approach is based on the analysis of digital gene expression and is capable of generating millions of short sequences [7,8]. While the second-generation sequencing technology can provide unprecedented levels of detail, one of its main challenges is the unavailability of intuitive, publicly available universal tools that can retrieve, process and visualize large amounts of generated data in a single package. Hundreds of computational programs for analysis of massive quantities of short reads are already available in the public domain [9]. These programs were designed either for read alignment [10,11], analysis of alternative splicing and gene expression [12,13] or visualization [14-16]. Our contribution with the presented work is creation of a package that leverages publicly available programs for next generation sequencing data analysis. This package aligns “reads” (or cDNA sequences), quantifies gene expression (in terms of RNA level) and visualizes results in a simple flow of linear data analysis.

Interpretation of second-generation sequencing data requires expertise in quality control, alignment of reads, quantitation of expression and visualization of massive volumes of data. These steps are commonly embedded in custom-made data analysis pipelines that consist of several steps and typically use some of the aforementioned programs. The data analysis process is performed in a specific order in which output of one operation is used as an input for the next one. Such an approach is highly non-trivial and may still present a challenge for sequencing facilities significantly smaller in size and resources than the National Human Genome Research Institute-funded large genomic centers [17]. To meet this challenge, we have developed Transcriptome Analysis with Circos (TrAC), a novel tool for comparative analysis and visualization of short reads based on Circos [16]. TrAC is a highly customizable RNA-Seq tool applicable for global transcriptome analysis with the additional feature that the visualization step deviates from the “one-gene-at-a-time” paradigm commonly practiced by the popular genome browsers [14,15]. With TrAC users can visualize whole pathways, cycles within pathways or linear segments of genes in any given configuration. We applied this tool to study energy metabolism in tissues with high-energy requirements such as normal brain and cancer cell lines.

## Results and discussion

We analyzed genes that encode enzymatic components involved in essential steps within metabolic pathways. There were 8 main enzymes (and their encoding genes) involved in the TCA cycle, and 10 main enzymes and genes in glycolysis (Table 1). As illustrated in standard biochemistry books, all the enzymes and substrates that participate in the TCA cycle and glycolysis have been known for a long time. However, we could find no definitive information on gene expression, at transcript level, for any of the genes involved in each of the two pathways. For example, for *HK1*, which encodes hexokinase 1, the entry step in glycolysis, the National Center for Biotechnology Information Reference Sequence (RefSeq) recognizes 5 alternatively spliced variants (NM_000188.2, NM_033497.2, NM_033498.2, NM_033500.2, NM_033496.2). For the purpose of visualization and to avoid overcrowding in the figures, we choose to present the longest transcripts only; i.e., NM_033500.2 for *HK1*. The mRNA sequence of each presented gene was selected in a similar manner from RefSeq database [18,19].

**Table 1:** Core genes of the TCA cycle and glycolysis

Gene name	Gene ID	mRNA accession number	Pathway	Copy Number of Transcript	
				
				UHRR	HBRR
*CS* (*Citrate synthase*)	1431	NM_004077	TCA cycle	11.2	7.4
*ACO2* (*Aconitase 2*)	50	NM_001098	TCA cycle	7.8	20.7
*IDH2* (*isocitrate dehydrogenase 2*)	3418	NM_002168	TCA cycle	13.1	6.2
*OGDH* (*oxoglutarate dehydrogenase*)	4967	NM_001165036	TCA cycle	2.8	3.3
*SUCLA2* (*succinate-CoA ligase*)	8803	NM_003850	TCA cycle	1.1	2.1
*SDHA* (*succinate dehydrogenase complex*, *sub. A*)	6389	NM_004168	TCA cycle	20.5	15.4
*FH* (*fumarate hydratase*)	2271	NM_000143	TCA cycle	6.5	2.8
*MDH1* (*malate dehydrogenase 1*)	4190	NM_005917	TCA cycle	10.4	26.2
*HK1* (*hexokinase 1*)	3098	NM_033500	Glycolysis	2.3	3.0
*GPI* (*glucose-6-phosphate isomerase*)	2821	NM_000175	Glycolysis	11.9	10.3
*PFKL* (*phosphofructokinase*, *liver*)	5211	NM_002626	Glycolysis	21.7	10.3
*ALDOA* (*aldolase A*, *fructose-bisphosphate*)	226	NM_000034	Glycolysis	49.9	25.0
*TPI1* (*triosephosphate isomerase 1*)	7167	NM_001159287	Glycolysis	68.4	29.8
*GAPDH* (*glyceraldehyde-3-phosphate dehydrogenase*)	2597	NM_002046	Glycolysis	886.6	287.3
*PGK1* (*phosphoglycerate kinase 1*)	5230	NM_000291	Glycolysis	67.4	15.1
*PGAM1* (*phosphoglycerate mutase 1*)	5223	NM_002629	Glycolysis	41.5	27.6
*ENO1* (*enolase 1*)	2023	NM_001428	Glycolysis	264.2	51.3
*PKM2* (*pyruvate kinase*)	5315	NM_002654	Glycolysis	37.7	21.3

### RNA-Seq data processing

**Figure 1:**
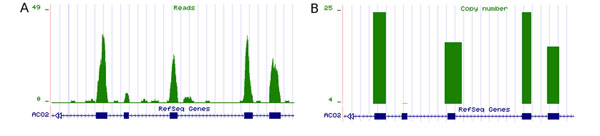
**RNA-Seq data representation** RNA sequencing reads coverage of five exons of the aconitase *ACO2* gene. Exons are blue bars; reads are presented in green. A Digital transcript topology generated by accumulation of every read mapped within a specific region (exon, intron, or transcript). B Absolute copy number provided a means to quantity transcribed products in the sample.

Reads generated on the next-generation sequencing platform from brain and cancer samples were aligned against elements from the human RefSeq database. In our read mapping with Bowtie [10], we imposed default parameters with two mismatches within the first 28 nucleotides (nt) of each read relative to the reference. From aligned short sequences, we calculated the digital expression signal by determining the number of mapped reads at each base on the reference. Visualization of the digital signal in the form of a histogram allowed us to investigate transcript topology. In particular, we were able to study the differences in sequencing coverage within the whole transcripts, genes, and exons (Figure 1A).

In order to provide biological meaning for the reads mapped on the reference and to report the digital expression signal as a scalar value, for each gene we considered both the number of mapped reads and the mapped length in base pairs. To quantify this expression, the ratio between mapped reads and transcribed length was appropriately rescaled with the total number of mapped reads and the total mapped transcriptome size, as previously described [20]. An example of the raw read count is shown in Figure 1A, while estimates of expression of each exon are shown in Figure 1B. Under the assumption of uniform read coverage, this copy number of transcripts corresponds to the amount of every expressed RNA present in the sample and is directly proportional to the RPKM (Reads mapping to the genome Per Kilobase of transcript per Million reads sequenced) value [20].

### The TCA cycle analysis

**Figure 2:**
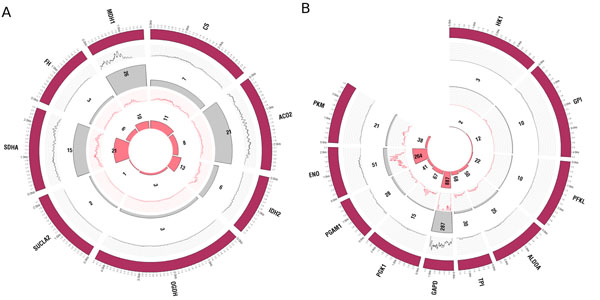
**Metabolic pathways** Main genes (maroon) of the TCA cycle (A) and glycolysis (B). The brain sample is shown in grey; the cancer sample is shown in pink. Copy number of each transcript is shown as a bar. The height of the bar corresponds to the quantity of the transcript present in the sample. Digital transcript topology for each gene shown as ‘wiggles’.

Gene expression values across samples were noticeably different. In the brain tissue, the trend in the first three steps of the TCA cycle, which involves the citrate synthase gene *CS*, aconitase *ACO2* and the isocitrate dehydrogenase *IDH2* gene, was qualitatively and quantitatively distinct relative to the trend observed in cancer. In particular, the number of transcribed copies of *ACO2* was higher than the numbers of *CS* and *IDH2*; however, in the cancer sample the situation was quite the opposite with copy numbers of *CS* and *IDH2* exceeding *ACO2* expression. We also noticed that the ratio of expression level of malate dehydrogenase *MDH* to fumarate hydratase *FH* in the brain sample was 8.6 while in the neoplastic sample it was 1.6 (Figure 2A). Previous work on yeast indicates that mutations in isocitrate dehydrogenase affect DNA instability [21]. For humans, a drastic change in *CS* expression, which may be further stimulated by excess concentration of Zn2+ ions and leads to changes in the local pH, has been observed in prostate carcinogenesis [22,23].

Visualization of digital expression demonstrated that sequence coverage by transcriptomic tool RNA-Seq is non-uniform but exhibits a highly reproducible pattern across different samples, as shown in Figure 2. The patterns were especially obvious in the case of *ACO2* and *MDH1* in the TCA cycle. In both samples, the *ACO2* and *SDHA* genes were well covered with reads along the whole length of the transcripts. However, the *MDH* coverage was biased towards the 5’ end and the middle of the transcript, whereas very few reads mapped to the 3’ end (Figure 2A).

### Glycolysis analysis

Every gene in the glycolysis pathway showed stronger expression in neoplastic cells than in the brain. The absolute transcript numbers of genes in both samples followed a similar pattern within first five steps in the pathway: the expression level gradually increased. However there were several steps in glycolysis that had very high levels of expression in both types of cells. In particular the digital signal for *GAPDH* and *ENO* were considerably higher in both samples when compared to other genes in the pathway. Furthermore, in cancer cell lines the copy numbers of *GAPDH* and ENO were 3 and 5 times higher than in brain tissue, respectively (Figure 2B).

### Discussion

**Figure 3:**
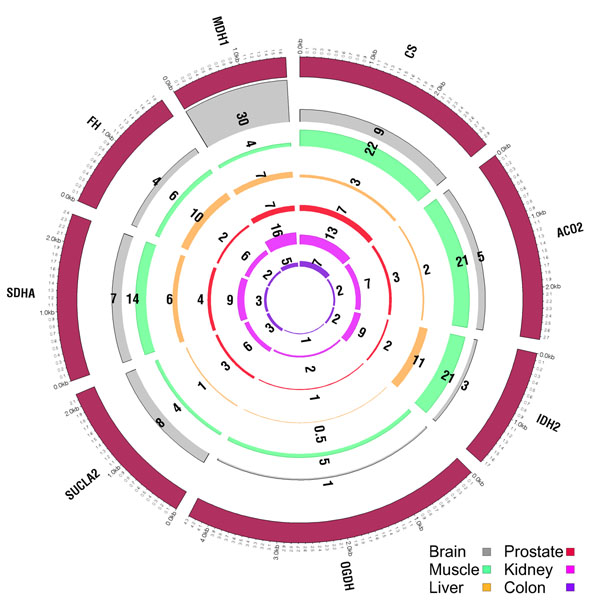
**The TCA cycle across different tissues** Gene expression values of the TCA cycle genes across six different tissues from the Human BodyMap Project by Illumina.

As expected, we showed profound differences in activities of genes involved in two major metabolic pathways: the TCA cycle and glycolysis. The analyzed samples, brain and cancer cell lines, were of particular interest because of their similar high-energy requirements. We also showed remarkable differences in the baseline expression level for genes involved in the TCA cycle and glycolysis across different normal tissues (Figure 3). Illumina, Inc (San Diego, California, U.S.) under the BodyMap Project sequenced these tissues.

Our approach revealed both quantitative signals for digital expression and topology of transcript coverage for all analyzed genes. The next generation mRNA sequencing provides an unbiased view of complete transcriptomes. To begin to make sense of transcriptomes represented by large and growing volumes of sequencing data, we need to be able to focus on individual subsets of genes. Topologically speaking, every pathway consists of one of the two major building blocks: a closed loop (such as the TCA cycle) and a linear cascade (such as glycolysis). The tool that we present, Transcriptome Analysis with Circos or TrAC, is our version of an unbiased approach to data analysis because it allows end users to pursue both hypothesis-driven and hypothesis-generating research. In hypothesis- driven analyses, the genes that participate in the major pathways are mainly known. TrAC is also suitable for hypothesis-generating research because an arbitrary list of genes that represent substructures of transcriptional networks can be very efficiently quantitated and visualized. This is a significant advantage over popular genome viewers such as Integrated Genomics Viewer, developed at the Broad Institute, that is constrained to visualize a single gene or a contiguous stretch of DNA on the same chromosome, but by default cannot quantitate gene expression or be used for differential expression. TrAC, therefore, represents a shift in NGS data analysis because it can do both. Moreover it is very flexible about its input, as it simply requires a list of fastq files and a list of genes of interest for analysis.

To appreciate the discovery potential of TrAC, we examined two major, extremely well-studied carbohydrate metabolic pathways for which we still have a very limited understanding of the transcriptional control and consequences of gene activity.

The differences that we observed in *ENO* expression in cancer cell lines versus brain support the hypothesis that *ENO* plays a role in pyruvate channeling towards the TCA cycle in mitochondria [24]. On the other hand, with the exception of breast, head and neck, and bone marrow cancers, *ENO* tends to be overexpressed in a majority of cancers [3]. With the mitochondrial aconitase gene, *ACO2*, it is quite the opposite as it is generally overexpressed in a small number of cancers such as melanoma and lung cancers [3]. In the P3 prostate cancer cell line, inhibition of *ACO2* did not cause a major shift in ATP production, but inhibition of both glycolysis and respiration were necessary to decrease the ATP content [25]. From a bioinformatics standpoint, the *ACO2* transcript is of particular interest because a microarray probe on the human Illumina gene arrays cannot be mapped using stringent mapping criteria (MAQC) since the probe has two mismatches relative to the reference. On the other hand, two mismatches on a 75bp-long read in next generation mRNA sequencing experiments are less of a problem because large numbers of reads do not all have to be of superb quality to provide confidence about gene expression.

Biological interpretation of the results presented would require experimental design beyond the scope of this study. In the absence of a carefully designed experiment to study changes in a global gene expression network due to manipulation of the main genes in the TCA cycle and glycolysis, we can attempt to understand the gene activity associated with these two processes through visualization with TrAC. While the objective of the work presented was not to explain the long-standing puzzle of the Warburg effect, the described package, TrAC, represents a viable tool for guiding the analysis. We expect that TrAC will simplify data analysis for non-bioinformaticians (manuscript in preparation). As discussed, TrAC can be scaled up to hundreds of genes involved in metabolic pathways; and we propose it as a bioinformatics tool for a simplified multi-gene rather than single-gene approach in biological problems.

## Conclusions

Next-generation sequencing technology was used to study gene expression/activity differences in metabolic pathways between normal and neoplastic cells. For this study we developed and implemented TrAC – Transcriptome Analysis with Circos, a novel computational data analysis and visualization tool. With the TrAC pipeline, we were able to process RNA-Seq data and from large volumes of sequencing data extract meaningful insights into a biological problem. Transcript copy numbers of the main genes involved in carbohydrate metabolism in brain and cancer cells were estimated and different gene expression patterns within two samples were revealed. We expect that this analytical tool will provide further insights into the subtleties related to the causality of the Warburg effect and neoplastic transformation. Our future research will involve translational investigation of the influence of described variations on the rates and qualities of ATP production in normal and neoplastic cells. Although we focused on the genes involved in the main steps of the TCA cycle and glycolysis, the developed approach is global and pathway-independent. TrAC is fully customizable and allows end-users to study any gene expression-related biological question (manuscript, in preparation).

## Methods

### Samples

Analysis of energy metabolism was performed on high quality RNA samples of normal brain and cancer cell lines. The brain sample consisted of FirstChoice Human Brain Reference RNA (HBRR by Ambion, cat #AM6050), pooled from multiple donors and several brain regions. The cancer sample was the Universal Human Reference RNA (UHRR by Stratagene), composed of RNA from 10 human cell lines [26]. These samples were sequenced by Illumina using the Genome Analyzer platform for the Sequencing Quality Control project, in a follow up to a large FDA-led Microarray Quality Control (MAQC Consortium) project [27]. In particular, we used 46.8 million HBRR and 50.6 million UHRR in 50 nt-long single-end reads.

### A pipeline for analysis and visualization of RNA-Seq data

The analysis of RNA-Seq data consisted of several steps: pre-processing, alignment, post-processing, and visualization. The pre-processing step consists of preparing reads for the alignment. The information content of each unique read was calculated according to the Shannon entropy formula (manuscript in preparation). The rationale for this filtering was our finding that the aligner tends to spend most of the CPU time aligning uninformative reads such as mononucleotide repeats from polyA tails or dinucleotide simple repeats; so filtering out such reads prior to the mapping significantly improved run times. Second, reads were sorted in an alphabetical order that facilitated the alignment process by providing the aligner with efficient data structure. After this initial step in the data analysis, the high information content unique reads were aligned with Bowtie [10], a freely available, memory- efficient and very fast short-read alignment software package. We aligned against the RefSeq [19] release downloaded in October of 2010 with a query to the NCBI web site as previously described [27]. We allowed up to two mismatches in the alignment. Mapped reads were saved in sorted SAM format that was used for gene expression quantitation estimates and post-processing visualization. We plotted the pileup vectors of digital expression for each expressed transcript to visualize transcript topology and read coverage. The number of reads and the covered transcript length were further used to estimate the number of transcribed mRNA copies of genes involved in main steps of cell metabolism. These results were finally visualized using the free software package Circos [16].

### Data analysis

In order to study topology of the transcripts, genes, or exons for reads aligned on the RefSeq reference, we generated a pile-vector for each transcript. This vector consisted of an integer count of mapped read coverage at each nucleotide position in a reference. Visualization of pile-vector allowed us to investigate the different features of transcript regions that we were interested in.

For every gene the estimated copy number (digital gene expression) was calculated by taking into account the number of mapped reads and the length of the transcript, appropriately rescaled with the total number of mapped reads and the total length of the sequenced transcriptome [20]:

where *N_Gene_* is a number of mappable reads aligned on the gene’s exons; *L_Gene_* is the sum of the gene’s exon lengths; *N_Transcriptome_* is the total number of mappable reads in the experiment; *L_Transcriptome_* is the length of the transcriptome.

## Authors' contributions

AAM worked on data analysis, processing, visualization and implementation of the TrAC tool. DH worked on data analysis, processing, and visualization. Both authors wrote the manuscript.

## Competing interests

The authors declare that they have no competing interests.
